# Macrocyclic poly(*p*-phenylenevinylene)s by ring expansion metathesis polymerisation and their characterisation by single-molecule spectroscopy†
†Electronic supplementary information (ESI) available. See DOI: 10.1039/c7sc03945j


**DOI:** 10.1039/c7sc03945j

**Published:** 2018-02-13

**Authors:** Benjamin John Lidster, Shuzo Hirata, Shoki Matsuda, Takuya Yamamoto, Venukrishnan Komanduri, Dharam Raj Kumar, Yasuyuki Tezuka, Martin Vacha, Michael L. Turner

**Affiliations:** a The School of Chemistry , The University of Manchester , Oxford Road , Manchester , M13 9PL , UK . Email: michael.turner@manchester.ac.uk; b Department of Materials Science and Engineering , Tokyo Institute of Technology , Ookayama 2-12-1, Meguro-ku , Tokyo 152-8552 , Japan . Email: vacha.m.aa@m.titech.ac.jp; c Division of Applied Chemistry , Faculty of Engineering , Hokkaido University , Sapporo , Hokkaido 060-8628 , Japan

## Abstract

Ring expansion metathesis polymerisation (REMP) has proven to be a viable approach to prepare high purity cyclic polymers. Macrocyclic polymers with a fully conjugated defect free backbone are of particular interest as these polymers have no end groups that can act as charge traps. In this work soluble macrocyclic poly(*p*-phenylenevinylene)s (***c*PPV**s) have been prepared directly *via* the REMP of substituted paracyclophanedienes. Single-molecule spectroscopy of the two topological forms of PPV *i.e.*, linear (***l*PPV**) and cyclic (***c*PPV**) revealed that ***l*PPV** exists in an extended conformation whereas the ***c*PPV** adopts a restricted ring-like conformation. Despite such large differences in the chain conformation, the spectral properties of the two compounds are unexpectedly very similar, and are dominated by torsional deformations in relatively short conjugated segments.

## Abstract 2

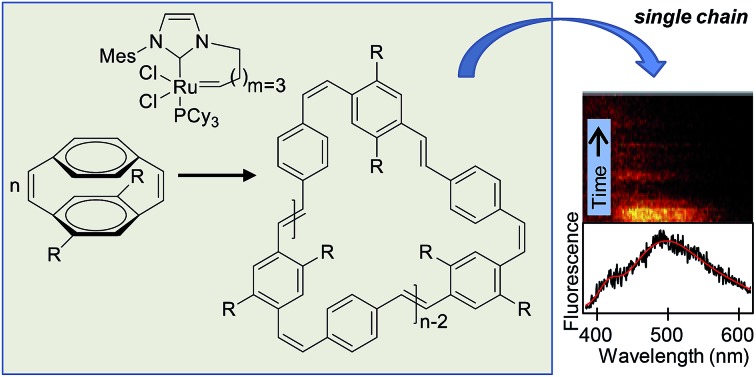
Ring expansion metathesis polymerisation (REMP) has proven to be a viable approach to prepare high purity macrocyclic phenylenevinylene polymers.

## Introduction

Macrocyclic chromophoric structures are essential components of the light-harvesting antenna of photosynthetic systems.^1^ To understand the operation of these natural chromophoric systems there has been considerable effort towards the synthesis or self-assembly of model systems based on porphyrins, perylenes,^2^–^4^ macrocyclic arylenes (phenyl, thiophene and pyridine) or phenylene-ethynylenes.^5^ The photophysical properties of these systems have often been characterized by single-molecule fluorescence detection and spectroscopy, which provides exceptional insight into the static and dynamic heterogeneity of the optical properties.^6^–^9^ Similar studies have helped to unravel the excitonic structure of the photosynthetic bacteria antenna^6^ and map the exciton localization dynamics in conjugated macrocycles.^8^ In both natural and artificial macrocycles with large exciton delocalization, the optical transitions to the lowest energy level are symmetry forbidden. This is in strong contrast to linear chains of head-to-tail arrangement in which the transitions to the lowest energy level are the most intense ones and carry all the oscillator strength, resulting in markedly different photophysics between the cyclic and linear molecular assemblies. In synthetic polymer chemistry there has been a continuous effort to synthesize ever more complex cyclic polymer topologies^10^ as the physical properties of these materials are very different to those of their linear counterparts, such as enhanced diffusion in solutions^11^ or melts,^12^ or micelle-forming ability and stability.^13^

Cyclic conjugated polymers represent a class of cyclic polymers with infinitely long π-conjugated systems with no end groups.^14^–^16^ These molecules are expected to increase the effective conjugation length over the linear analogues and show a more constricted excited state geometry, reduced chain–chain interactions in solution due to less chain entanglement and exhibit greater extinction coefficients. The end groups in linear conjugated polymers are known to act as charge trapping sites and as a source of degradation pathways in OPV and OFET performance.^17^,^18^ In addition, cyclic polymers are potentially advantageous over long linear chains in applications that require effective interaction between entities moving along the same main chain, such as triplet excitons in triple-triplet annihilation-based upconversion schemes where exciton collisions are necessary, or the recombination of charges in OLEDs. Small ring systems such as cycloparaphenylenes exhibit unexpected photophysical properties due to their size, symmetry and conformations.^19^

There are two main approaches known for the preparation of cyclic polymers,^20^,^21^ the cyclisation of telechelic polymers under high dilution,^22^ and the ring expansion metathesis polymerisation (REMP) first reported by Grubbs *et al.*^23^,^24^ There are examples of cyclic π-conjugated oligomers reported including [*n*]cycloparaphenylenes by Bertozzi,^25^ Itami^26^ and Yamago,^27^ cyclo[*n*]thiophenes by Bäuerle,^28^,^29^ macrocyclic oligo(fluorene)s by Müllen^30^ and a templated approach towards porphyrin nanorings by Anderson.^31^ Recently, Veige^32^ reported a direct catalytic approach to monosubstituted cyclic polyacetylenes by the polymerisation of terminal alkynes using tetraanionic tungsten complexes.

**Scheme 1:**
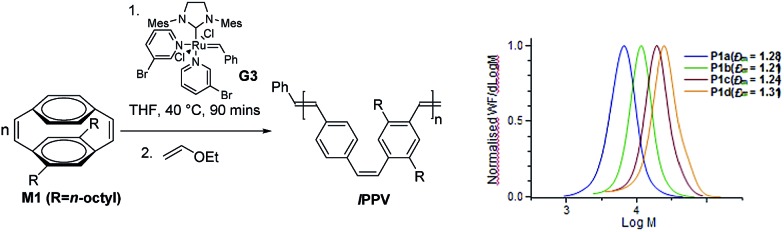
ROMP of cyclophanediene monomer **M1** towards soluble linear poly(*p*-phenylenevinylene)s.^40^

Poly(*p*-phenylenevinylene)s (PPVs) have been extensively studied for their semiconducting and optical properties ever since Holmes *et al.* published the use of the unsubstituted PPV in the first polymer-based light emitting diode.^33^,^34^ Most chain growth synthetic routes to these materials proceed by uncontrolled mechanisms and lead to poor control of the *M*_n_, broad *Đ*_m_, poorly defined end groups and the inclusion of backbone defects.^35^ Control of the polymerisation process can be achieved by the ring opening metathesis polymerisation (ROMP) of strained [2.2]paracyclophanedienes^36^–^41^ and [2.2.2]paracyclophanetrienes.^42^ For example it has been shown that ROMP of substituted [2.2]paracyclophanedienes proceeds by a well controlled living polymerisation in which the target linear polymers are obtained with predetermined *M*_n_, narrow *Đ*_m_, controlled end groups and a defect free backbone consisting solely of alternating phenylene rings and vinylene bonds (Scheme 1).^40^

In light of the potential advantages of conjugated cyclic polymers over the linear analogues for electronic applications we are investigating the use of novel synthetic methods to deliver truly macrocyclic conjugated polymers. Herein we report the synthesis of cyclic poly(*p*-phenylenevinylene)s (***c*PPV**) by the REMP of paracyclophanedienes and single-molecule fluorescence spectroscopy of individual chains of ***c*PPV** as well as its linear counterpart (***l*PPV**) of the same molecular weight. The conformations of single ***c*PPV** and ***l*PPV** chains were determined by measuring the absorption (excitation) anisotropy^43^–^45^ and model calculations. These measurements were complemented by recording the single chain emission anisotropy^45^,^46^ as single chain fluorescence spectra provide information on the size of the conjugated segments and on the amount of intrachain structural disorder. The comparison of the optical properties of the cyclic (***c*PPV**) and linear (***l*PPV**) structures provides a greater understanding of the relationship between conformation and spectra in these conjugated polymers.^47^–^53^

## Results and discussion

### Synthesis of cyclic phenylenevinylenes (***c*PPV**)

**Scheme 2:**
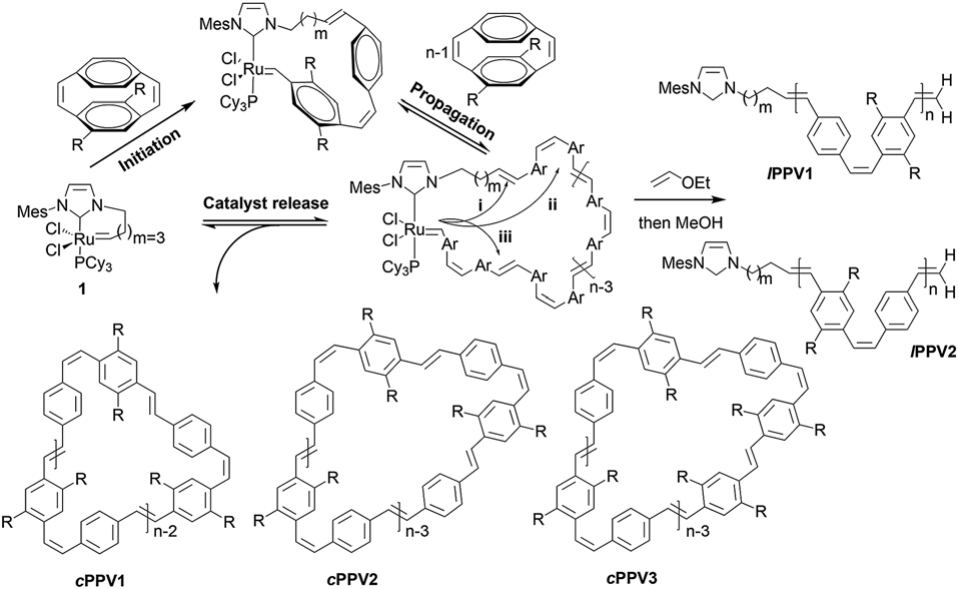
REMP of substituted cyclophanedienes **M1** and **M2** to give macrocyclic poly(*p*-phenylenevinylene)s (***c*PPV**).

The REMP of cyclophanedienes, **M1** (R = *n*-octyl)^54^ and **M2** (R = *O*-2-(*R*/*S*)-ethylhexyl),^55^ using the cyclic ruthenium alkylidene complex **1** to give soluble cyclic poly(phenylenevinylene)s (***c*PPV**) is shown in Scheme 2. The cyclophanedienes were synthesized by an adaption of the previously reported synthesis^52^,^53^ and complex **1** by the method of Fürstner *et al.*^56^

***c*PPV**s are generated by ring extrusion through the secondary metathesis of the initially formed metallacycle between the ruthenium benzylidene moiety and a vinylene bond of the polymer backbone. As this is a random process and the monomers **M1** and **M2** are asymmetric several pathways are possible, and these are shown as (i)–(iii) in Scheme 2. In the ideal situation, pathway (i), metathesis occurs on the vinylene bond adjacent to the NHC ligand of the ruthenium carbene, resulting in regeneration of the original catalyst and generating a cyclic polymer with the number of repeat units equal to the degree of polymerisation. In pathway (ii) metathesis occurs at a point in the polymer backbone close to the NHC ligand, resulting in a small ruthenium benzylidene containing metallacycle and a large cyclic PPV polymer. In pathway (iii) metathesis occurs at a vinylene bond of polymer backbone closer to the ruthenium centre, resulting in a large cyclic ruthenium benzylidene and a small PPV oligomer. In this pathway several oligomeric PPVs could be produced if the metallacycle was large enough. As *cis*-vinylenes are more reactive towards metathesis and the monomers **M1** and **M2** are asymmetric the formation of several cyclic species is possible; ***c*PPV1** with the number of repeat units equal to *n*, ***c*PPV2** with an additional unsubstituted phenylene ring and ***c*PPV3** with an additional substituted phenylene. In all cases the polymers are isolated with an alternating *cis*–*trans* vinylene backbone as only one bond of the cyclophanediene is subjected to metathesis (Scheme 2 and Fig. S4†).

REMP of the dialkyl monomer **M1** using initiator **1** in C_6_D_6_ (0.1 M) at 60 °C ([**M1**] : [**1**] = 10) was almost complete (monomer conversion >96%) after 2 h of reaction. The polymerisation reaction was followed by *in situ*^1^H NMR spectroscopy and major signals were observed that correspond to the formation of the desired poly(phenylene-2,5-dioctyl-phenylenevinylene) with only a trace of unconsumed monomer at *δ* 6.19 ppm (Fig. S1 and S4†).

**Fig. 1:**
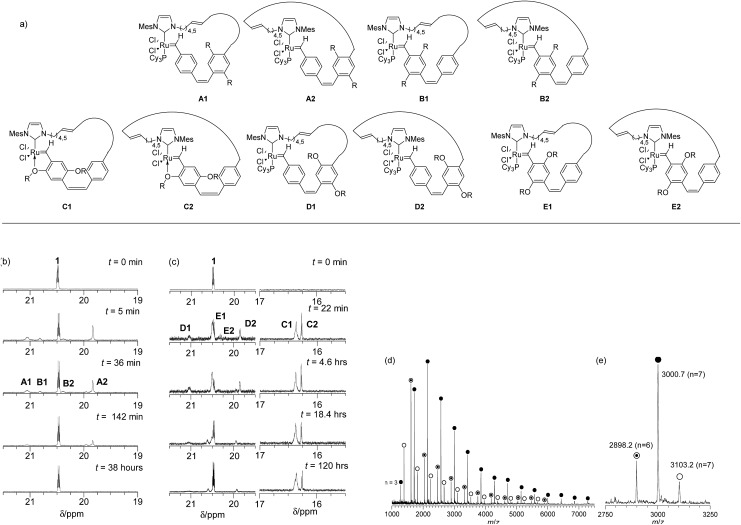
(a) Metallacyclic intermediates in the REMP of monomers **M1** and **M2** (top figure) (b) time dependent ^1^H NMR spectrum (C_6_D_6_) of REMP of monomer **M1** with **1** in carbene region (bottom left) (c) time dependent ^1^H NMR spectrum (C_6_D_6_) of REMP of monomer **M2** with **1** in carbene region (bottom middle) (d) MALDI-TOF mass spectrum of purified cyclic polymer from REMP of monomer **M1**. The major series 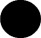 corresponds to cyclic polymers with an equal number of phenylene/substituted phenylene rings (***c*PPV1**) and the series 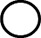 and 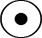 to cyclic polymers with an extra phenylene (***c*PPV2**) or substituted phenylene ring (***c*PPV3**). (e) Mass region 2750–3250 Da (see also Scheme 2).

Four different sets of carbene signals were observed in the ^1^H NMR spectrum (see Fig. 1b, *t* = 36 min) in addition to that of **1** (*δ* 20.43 ppm). These were assigned to metallacycles **A1** (*δ* 21.01 ppm), **B1** (*δ* 20.78 ppm), **B2** (*δ* 20.34 ppm) and **A2** (*δ* 19.79 ppm) as shown in Fig. 1a. The lower chemical shift of the carbene proton in intermediate **A2** over that of **A1** is due to the shielding effect of the mesitylene moiety of the NHC ring.^57^ The catalyst is regenerated by ***c*PPV** extrusion in pathway (i) (see Fig. 1b and S2†) and **1** is the only carbene species observed at long reaction times (>38 hours). However the total amount of ruthenium carbene species present at the end of the reaction was only 39% of the initial concentration of **1** presumably due to decomposition by traces of oxygen present in the reaction mixture at such extended reaction times (Fig. 1 and S2†). After 38 h the reaction was quenched by adding ethyl vinylether and the polymer precipitated on a Celite plug by adding methanol. The polymer was collected by extraction of the precipitate with chloroform. The ^1^H NMR spectrum of the crude material showed characteristic signals for the desired poly(*p*-phenylenevinylenes) and small resonances for linear polymer end groups at 6.00–5.00 ppm for the vinyl and 2.30–1.75 ppm for the dihydroimidazole group (see Scheme 2 and Fig. S4†).

Purification by precipitation in MeOH reduced the intensity of the signals at both *δ* 6.00–5.00 ppm and *δ* 2.00–1.75 ppm indicating that these signals correspond to the end groups of oligomers (Fig. S4 and S6†). The molecular weight of the isolated polymers was determined by GPC at the end of the reaction. These were significantly higher (*M*_n_ > 7 kDa, see Table S1†) than those expected for the initial [**M1**] : [**1**] ratio (4.3 kDa) and a well-controlled polymerisation.

Increasing the [**M1**] : [**1**] from 10 to 20 resulted in an increase in the molecular weight (*M*_n_ > 13 kDa) in line with the doubling expected. There was no correlation between the molecular weight of the polymer and the initial concentration of **M1** (0.1 to 0.025 M, Table S1†) but reactions at lower temperature (50 °C) in THF solvent gave polymers with lower dispersities (*Đ*_m_ = 1.8) due to reduced rates of secondary metathesis. MALDI-TOF mass spectrometry of the polymers after precipitation into methanol suggested that the major product is the linear polymers. This contradicts the low intensity of the end group signals in the ^1^H NMR spectra (see Fig. S5 and Table S2†) and it appears that the linear polymers with polar end groups are ionized far more readily and are observed preferentially.^58^,^59^

Trace linear contaminants were removed by column chromatography on silica, eluting the desired ***c*PPV** with a toluene/hexane (20/80) solvent mixture (33% yield). The MALDI-TOF mass spectrum of the purified material showed three major cyclic polymer series: ***c*PPV1** (*e.g. m*/*z* = 3000.7, *n* = 7), ***c*PPV2** (*e.g. m*/*z* = 2998.2, *n* = 6) and ***c*PPV3** (*e.g. m*/*z* = 3103.2, *n* = 7) (see Fig. 1d and Table S2†). The presence of these three major series for the ***c*PPV** is consistent with no control of the regiochemistry during secondary metathesis that leads to ring extrusion (see Scheme 2).

**Fig. 2:**
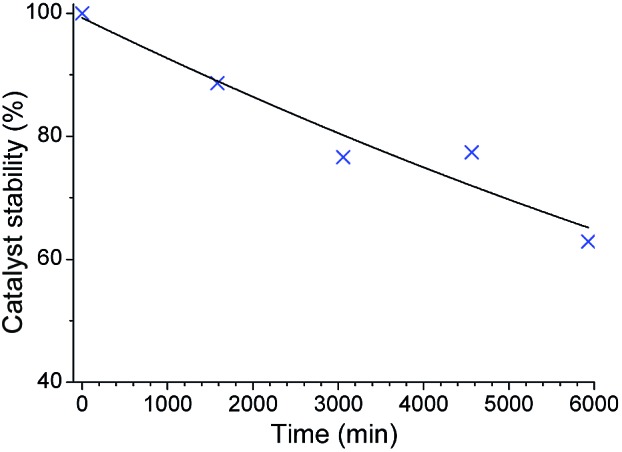
Stability of the catalyst **1** in C_6_D_6_ at 60 °C.

The REMP of the alkoxy substituted monomer **M2** was initiated by **1** in C_6_D_6_ (0.1 M) at 60 °C ([**M2**]/[**1**] = 9) and followed by ^1^H NMR spectroscopy (Fig. S8†). Complete monomer conversion was observed after 18 h of reaction at 60 °C and new sets of carbene signals were observed at *δ* 21.17 (**D1**), 20.59 (**E1**), 20.49 (**E2**), 19.92 (**D2**), 16.46 (**C1**) and 16.37 ppm (**C2**) (see Fig. 1a and c). The intensities of these signals remained constant over reaction times up to 16 h and the signals at *δ* 21.17 and 19.9 ppm were observed in the REMP of both monomers **M1** and **M2** as they correspond to the carbene moiety attached to the unsubstituted phenyl ring in **A** (**1**, **2**) and **D** (**1**, **2**) (Fig. 2 and S8†).

It appears that the extrusion of the ***c*PPV** for alkoxy monomer **M2** was inefficient due to reduced rates of back-biting compared to that in alkyl monomer **M1** as the initiator can coordinate to the polymer backbone through the alkoxy oxygen atom.^57^ On extended reaction time (8 days and 13 days) a reduction in the intensity of the carbene signals was observed and an increase in the carbene signal at *δ* 20.45 ppm corresponding to the reformation of **1** was also detected. The reactions were quenched by addition of ethylvinyl ether and the polymers precipitated in methanol on Celite and isolated by extraction with hexane. The ^1^H NMR spectra of these isolated polymers suggested that the material is predominantly cyclic as only very weak signals for polymer end groups were observed. However, MALDI-TOF mass spectrometry always showed traces of linear contaminants and it proved impossible to isolate purely cyclic polymers from these reactions as the extrusion of the ***c*PPV** was so slow (see Fig. S10†).

### Photophysical and single molecule characterisation

The ***c*PPV** prepared using **M1** as well as their linear counterparts (***l*PPV**)^40^ were characterized using standard UV-vis absorption and photoluminescence spectroscopy. The ring-linear pairs of the same molecular weight and composition provide an excellent opportunity to study elements of the basic aspects of the relationship between a conjugated polymer chain conformation and its photophysical properties. For this purpose, single-molecule spectroscopy is a tool of choice because it can provide information both on the conformation and fluorescence spectra. The conformation-properties relationship has been a long-standing topic for the PPV family of conjugated polymers, and has been traditionally approached by conventional spectroscopy of solvent controlled modifications of cast films.^60^ The advent of single-molecule spectroscopy has brought new insights into the problem^50^ and resulted in unexpected discoveries. It has been, for example, reported that bending of a PV oligomer or alternatively bending of a conjugated segment within a PPV chain causes considerable broadening and red shift of fluorescence spectra.^47^ We, therefore, expect that the linear and cyclic PPV chains will have different conformations that reflect their different topology, and that these different conformations will give rise to dramatically different photophysical properties.

### UV-vis data of linear and cyclic PPVs

**Fig. 3:**
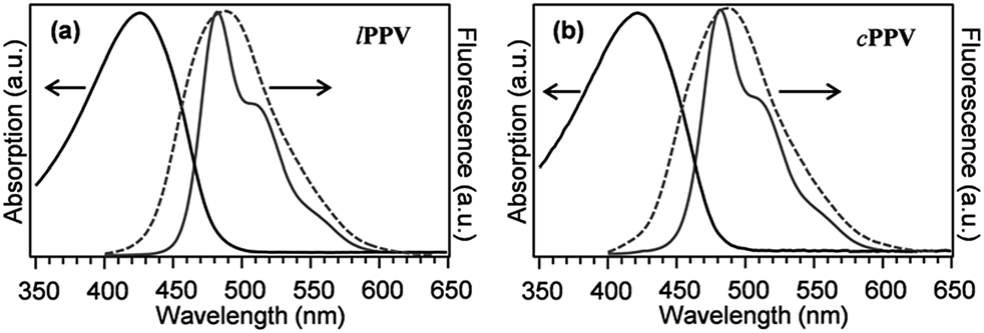
Absorption and fluorescence spectra of ***l*PPV** (a) and ***c*PPV** (b) measured in dilute toluene solutions at the concentration of 10^–6^ M (full lines) and fluorescence spectra in PMMA matrix (dashed lines). Fluorescence was excited at 375 nm.

Results of UV-vis characterisation of ***l*PPV** and ***c*PPV** in toluene solutions and in PMMA matrices are shown in Fig. 3. The absorption spectra are similar in shape and position, with the spectrum of ***c*PPV** slightly blue shifted (at 421 nm) from the ***l*PPV** (at 426 nm). The fluorescence spectra both in solution and in PMMA matrix are almost identical, with peaks at 484.4 nm and 485.0 nm in toluene and 488.0 nm and 488.2 nm in PMMA film for ***l*PPV** and ***c*PPV**, respectively.

### Single-molecule characterisation

Linear and cyclic PPV chains are expected to have different conformations due to their different topology. Experimentally, the conformations were characterised by measuring absorption (excitation) and fluorescence polarisation anisotropy on a statistical ensemble of single chains dispersed in an inert solid-state polymer matrix and by reconstructing the possible conformations by numerical simulations. The different conformations were then related to the photophysical properties of the PPV chains by measuring and analysing single-chain fluorescence spectra and their time evolution. The single-chain spectroscopic characterisation is further complemented by bulk experiments in which the PPV chains are doped into drop-cast PEMA films, the films are stretched to induce conformational changes of the PPV chains and spectral changes that reflect the conformation changes are measured.

### Absorption polarisation anisotropy

**Fig. 4:**
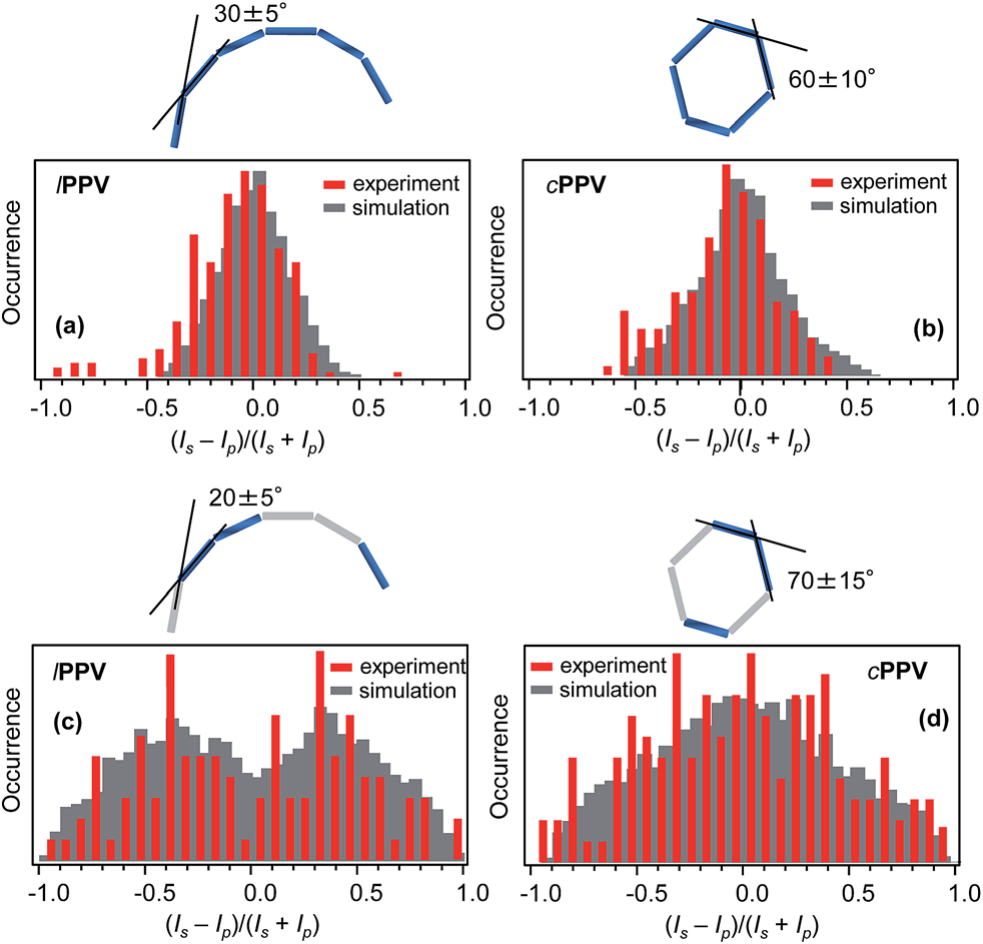
Top: experimental (red) and simulated (grey) distributions of the absorption (excitation) polarization anisotropy parameter *a*_A_ for ***l*PPV** (a) and ***c*PPV** (b). The schematic illustrations above the graphs indicate the projections onto the sample plane of the most probable conformations of the respective chains corresponding to the simulated distributions. Bottom: experimental (red) and simulated (grey) distributions of the fluorescence polarization anisotropy parameter *a*_F_ for ***l*PPV** (c) and ***c*PPV** (d). The schematic illustrations above the graphs indicate the projections onto the sample plane of the most probable conformations of the respective chains with 3 emitting conjugated segments. The conformations correspond to the simulated distributions.

The absorption anisotropy for each single molecule was evaluated by analysing the emission intensities *I*_s_ and *I*_p_ corresponding to two orthogonal linear polarisations s and p of the excitation laser and calculating the degree of the anisotropy as *a*_A_ = (*I*_s_ – *I*_p_)/(*I*_s_ + *I*_p_). The distributions of *a*_A_ for both ***l*PPV** and ***c*PPV** obtained for a statistical ensemble of 276 (***l*PPV**) and 136 (***c*PPV**) single molecules (Fig. 4a and b) are centered at 0 values and most of the data points are located between –0.5 and 0.5. The distribution of the ***c*PPV** is slightly sharper. The measured absorption anisotropy distributions are results of average conformations of the ***c*PPV** and ***l*PPV** chains. To reconstruct the conformations from the experimental data we performed numerical simulations. Assuming in analogy with other phenylenevinylene polymers that the conjugation length is approximately 6 monomer units^50^ the ***c*PPV** and ***l*PPV** chains are each modelled using 6 conjugated segments, each represented by a transition dipole moment. We have verified by the TD-DFT calculations that the transition dipole moments in the regularly alternating *cis*/*trans* chain are oriented approximately along the main axis of the polymer chain. In solution, the chains can undergo *cis*/*trans* isomerization (photo- and/or thermally induced). The resulting *trans*-segments also have transition dipoles oriented along the polymer chain. The chains are thus represented by 6 head-to-tail aligned transition dipole moments (rods). The numerical simulations (details of which can be found in ESI†) involved systematic generation of various conformations by varying the angles between the 6 consecutive segments. For each generated conformation a simulated histogram of the expected absorption anisotropy was calculated (Fig. S12†) and these simulated histograms were compared with the experimentally measured ones. Based on the best agreement between the measured and calculated histograms the most probable conformation of the ***l*PPV** chain was selected, as shown in Fig. 4a. For the cyclic polymer ***c*PPV**, the simulations have to account for the fact that the end of the last segment has to coincide with the beginning of the first segment. To satisfy this condition, the model of the ***l*PPV** was used for the first 3 segments, and the remaining 3 segments were constructed as a mirror image of the first three. The most probable conformation of the ***c*PPV** is shown in Fig. 4b.

### Fluorescence polarisation anisotropy

For each single chain the s and p polarised fluorescence intensities *I*_s_ and *I*_p_ were analysed and the degree of the anisotropy was calculated as *a*_F_ = (*I*_s_ – *I*_p_)/(*I*_s_ + *I*_p_). The distributions of *a*_F_ for both ***l*PPV** and ***c*PPV** obtained for a statistical ensemble of 108 (***l*PPV**) and 145 (***c*PPV**) single molecules are shown in Fig. 4c and d. Unlike the absorption anisotropy, the fluorescence anisotropy is markedly different between the cyclic and linear polymers. The *a*_F_ distribution for ***c*PPV** has a broad peak centered at 0 and spans values from –1 to 1. The distribution for ***l*PPV** shows a bi-modal structure with peaks at approximately –0.4 and 0.4, a minimum at 0 and with values spanning the whole range from –1 to 1.

The measured distributions were reproduced by numerical simulations (details can be found in ESI†). The simulations were done with the assumption that the emission originates from a limited number of conjugated segments due to efficient energy transfer and exciton localisation.^53^ The number of emitting segments were systematically decreased to 5, 4, 3 and 2 and in each chain the actual emitting segments were chosen randomly. Statistical comparison of the simulated and measured distributions (Fig. S13†) showed that the experiments are best reproduced with the emission from 3 conjugated segments for both ***l*PPV** and ***c*PPV** (Fig. 4c and d). The corresponding most probable conformations shown in Fig. 4 are very close to those obtained from the absorption anisotropy measurements and this result confirms the validity of this approach and reliability of the reconstructed conformations.

### Single chain spectra

**Fig. 5:**
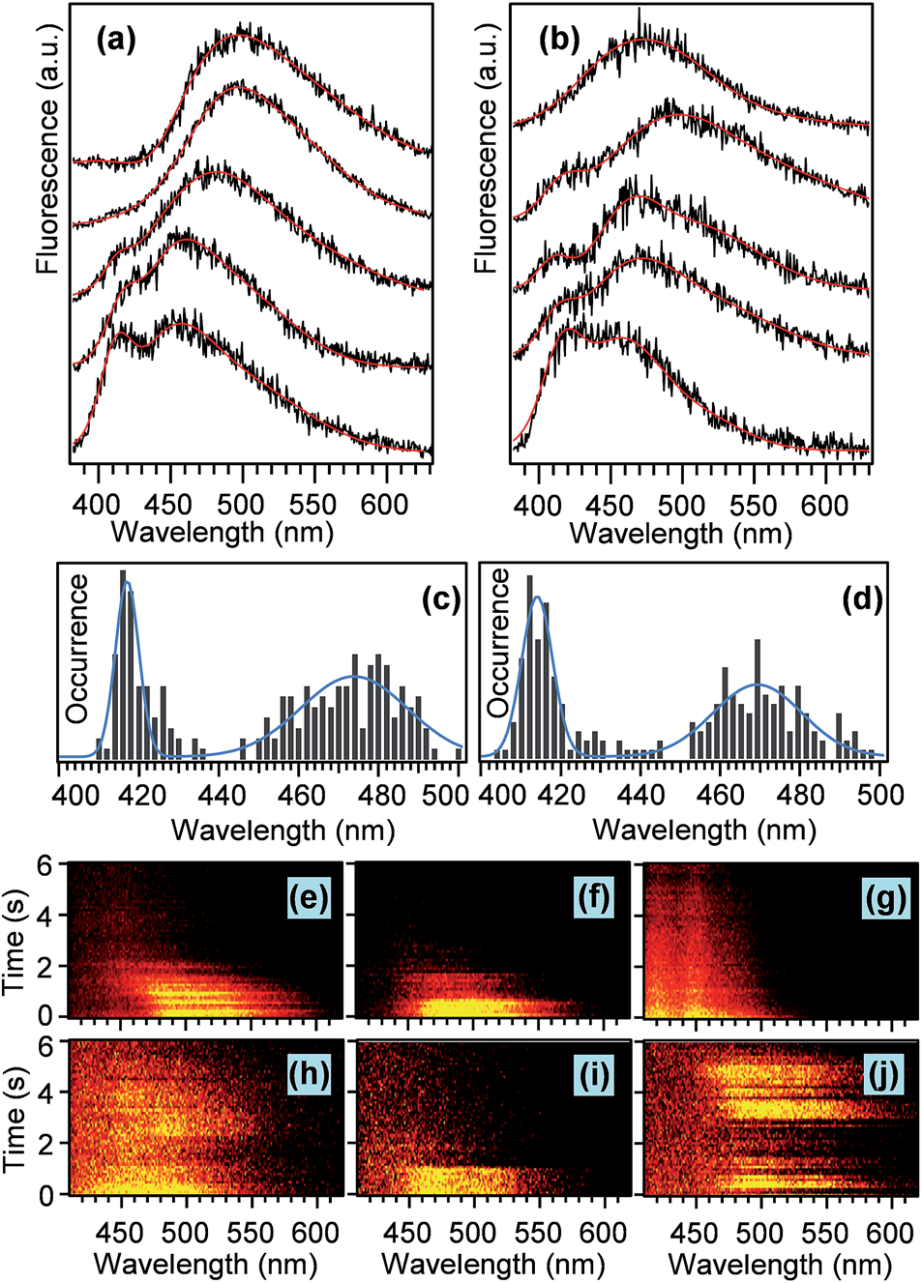
Typical single-chain fluorescence spectra of ***l*PPV** (a) and ***c*PPV** (b), and distributions of the spectral peaks of the main bands and short-wavelength shoulders obtained from Gaussian fitting of the single-chain spectra of ***l*PPV** (c) and ***c*PPV** (d). The panels (e–g) show typical patterns of time-evolution of single-chain spectra of ***l*PPV**, the panels (h–j) show the same for ***c*PPV**.

The different topology and the resulting conformations of the ***l*PPV** and ***c*PPV** are expected to result in dramatically different photophysical properties. To study the photophysical properties on single molecule level we measured and analysed fluorescence spectra of individual ***l*PPV** and ***c*PPV** chains. Representative examples of such spectra are shown in Fig. 5a and b. The spectra of both compounds are composed of a main band and a short-wavelength shoulder. The relative intensity of the shoulder varies from molecule to molecule. All spectra were fitted with a sum of three and in some cases four Gaussian functions and the spectral positions of the main band and of the shoulder were determined from the fits.

Results of the spectral analysis of 130 chains for each ***l*PPV** and ***c*PPV** are summarised in histograms in Fig. 5c and d. The short-wavelength shoulder is centered at 417 nm in ***l*PPV** and at 414 nm in ***c*PPV**.

The position of the main band is at 474 nm in ***l*PPV** and at 468 nm in ***c*PPV**. We have previously studied phenylenevinylene oligomers^48^ on single molecule level and observed different spectral bands corresponding to different conjugation lengths within oligomers of the same length. However, direct comparison with that work is not possible because of different stereochemistry and different nature of the substituents (alkoxy chains in ref. 48*vs.* alkyl chains in the current work) that causes spectral shifts of the main chain transitions.^61^ In analogy with previous work on unsubstituted PV oligomers in solution^62^ we may tentatively argue that the main band and the short-wavelength shoulder in both ***l*PPV** and ***c*PPV** correspond to conjugated segments that differ in the conjugation length by one monomer unit. The differences in the spectral properties of single chains between the ***l*PPV** and ***c*PPV** are surprisingly minor and include mainly the slightly shorter wavelengths of both the main bands (468 nm *vs.* 474 nm) and the shoulders (414 nm *vs.* 417 nm) for the cyclic ***c*PPV**. In addition, the fraction of the single chains that show the short wavelength shoulder is 1.4 times larger for the ***c*PPV**.

These observations reflect the fact that the cyclic structure of the ***c*PPV** restricts the available conformations of individual conjugated segments and of the chain as a whole. Such restrictions can cause both minor conformational differences that are manifested as the small blue shifts of the peaks in the spectral histograms, and larger topological defects that lead to the increased fraction of the shorter conjugated segments in ***c*PPV**. The single molecule emission intensity and spectra exhibit a variety of dynamic behavior, with examples shown in Fig. 5e–j. The chains of both compounds can show one-step (Fig. 5i), two-step (Fig. 5f) or continuous (Fig. 5g) photobleaching as well as blinking (Fig. 5j).

Approximately 10% of molecules of both compounds show continuous blue shift of the main band of the spectra in time (Fig. 5e). However, we have not observed large discrete spectral jumps as found before for conjugated oligomers^48^ and polymers.^63^ In a few chains of the cyclic ***c*PPV** we have also observed a slight red shift of the spectral main band in time (Fig. 5h). While a blue shift during photobleaching is a commonly observed phenomenon in conjugated polymers,^50^ a red shift is a rare event which could be related to the restriction on segmental conformation of the ***c*PPV**. In a spin-coated film the ***c*PPV** chains find themselves in highly strained state, the relaxation of which could lead to the red shift of the emission spectra.

### Bulk spectroscopic characterisation and DFT calculations

To get further insight into the nature of the observed spectral features and the conformational differences, the ***l*PPV** and ***c*PPV** were doped into free-standing PEMA films prepared by drop casting, and conformational changes and defects were induced by mechanical manipulation of the films. For that purpose, the films were heated above glass transition temperature and stretched to about twice of the original length. After cooling, fluorescence spectra and lifetimes of the stretched films were measured. Stretching of the films causes blue shift of the main band and an appearance of a short-wavelength shoulder (Fig. S14†). Both these effects are more prominent for the ***c*PPV** compound. In both ***l*PPV** and ***c*PPV**, fluorescence lifetimes show two components (0.9 ns and 6.8–6.9 ns). Spectrally, the shorter lifetime component is located in the region of the main fluorescence band whereas the longer component is blue-shifted and overlaps more with the short-wavelength shoulder (Fig. S14†). This large difference in lifetimes between the main band and the shoulder indicates that the origin of the two spectral features may involve other factors than only a difference in conjugation length, as assumed from the single-molecule spectra.

We further used TD-DFT calculations to reproduce the large distribution of the single-molecule spectral peaks within the main band, the short-wavelength shoulder and the fluorescence lifetimes. The calculations are based on the assumption that in solution at least part of the *cis* conformations in the alternating *cis*/*trans* chain undergo isomerisation (photo- or thermally induced), resulting in the presence of all-*trans* segments of different lengths. The calculations were done on linear units containing eight phenylenevinylene segments. Details of the calculations and examples of results are presented in ESI, Fig. S15.† Based on the calculation results, we assign the main fluorescence band in both ***l*PPV** and ***c*PPV** to longer all-*trans* segments with different amount of torsional defects as well as to shorter segments without such defects. Further, calculation including a rotation along a single bond in a *cis* state indicates that torsionally distorted *cis* conformations could contribute to the emission in the short-wavelength shoulder. The calculated smaller oscillator strength of these states would explain the longer fluorescence lifetimes observed in the short-wavelength shoulder for both ***l*PPV** and ***c*PPV**.

## Conclusions

The REMP of highly strained paracyclophanedienes gives macrocyclic poly(*p*-phenylenevinylenes). Alkyl substituted monomers (**M1**) gave purely cyclic polymers (***c*PPV**) as shown by NMR spectroscopy and MALDI-TOF mass spectrometry. By contrast the alkoxy substituted monomers (**M2**) gave pure metallacycles but ring extrusion to release cyclic ***c*PPV** was slow due to the complexation of the ruthenium centre by the oxygen of the alkoxy substituent. Hence cyclic polymers derived from **M2** always contained traces of linear contaminants. The conformation and photophysical properties of both the ***c*PPV** and ***l*PPV** topologies from **M1** were studied using single-chain absorption and fluorescence polarization anisotropy. The studies indicated that in solid-state matrices the ***l*PPV** exists in an extended conformation whereas the ***c*PPV** adopts a restricted ring-like conformation. Despite these differences in the chain conformation, the spectral properties of the two compounds are very similar, and are dominated by torsional deformations and influenced by distorted *cis* states. The similarity of the photophysical properties is an unexpected result as previous single-molecule studies indicated large differences in spectral characteristics due to conformational distortions, but such results can be understood and explained by a conjugation length that is shorter relative to the length scales that determine the conformations in both types of polymers.

## Conflicts of interest

There are no conflicts to declare.

## Supplementary Material

Supplementary information
