# Supplementary material for: Macrocyclic poly(p-phenylenevinylene)s by ring expansion metathesis polymerisation and their characterisation by single-molecule spectroscopy
Source: Chem Sci. 2018 Feb 13;9(11):2934–41. doi: 10.1039/c7sc03945j (PMC5915795; doi:10.1039/c7sc03945j)
Supplement: Supplementary file 1 [file SC-009-C7SC03945J-s001.pdf]

Electronic Supplementary Material (ESI) for Chemical Science.
This journal is © The Royal Society of Chemistry 2018

Supporting Information

Macrocyclic Poly(*p*-phenylenevinylene)s by Ring Expansion Metathesis Polymerization and their Characterization by Single-Molecule Spectroscopy

Benjamin John Lidster[a], Shuzo Hirata[b], Shoki Matsuda[b], Takuya Yamamoto[c], Venukrishnan Komanduri[a], Dharam Raj Kumar[a], Yasuyuki Tezuka[b], Martin Vacha*[, b], Michael L. Turner*[, a]

[a.] The School of Chemistry, The University of Manchester, Oxford Road, Manchester, M13 9PL, U.K.

[b.] Department of Materials Science and Engineering, Tokyo Institute of Technology, Ookayama 2-12-1, Meguro-ku, Tokyo 152-8552, Japan.

[c.] Division of Applied Chemistry, Faculty of Engineering, Hokkaido University , Sapporo, Hokkaido, 060-8628, Japan.

CONTENTS

General experimental details for synthesis S2

General experimental procedure for REMP S3

General experimental procedure for ROMP S3

REMP of alkyl monomer **M1** with catalyst **1** S4-7

REMP of alkyl monomer **M2** with catalyst **1** S7-8

Sample preparation and experimental setup for photophysical and single-molecule characterisation S9

Absorption polarisation anisotropy S9-11

Fluorescence polarisation anisotropy S11-13

Bulk spectroscopic characterisation S14

DFT calculations S14-16

References S16

General Experimental details for synthesis:

Nuclear magnetic resonance (NMR) spectra were obtained on either 400 MHz or 500 MHz Brucker spectrometers. Chemical shifts are reported in ppm relative to the indicated residual solvent (^{1}H NMR spectroscopy; 7.26 ppm for [*d*] chloroform and 7.16 ppm for [d_6]-benzene. The Longitudinal relaxation time constant (T1) for the paracyclophanediene monomers **M1**, **M2**, and Catalyst **1** were determined by inversion-recovery with a maximum T1 of 3 s, so relaxation delay of 15 s was used in all of the experiments. ^{13}C NMR spectroscopy; 77.16 ppm for [*d*]-chloroform, 128.06 ppm for [d_6]-benzene). The following abbreviations are used to indicate the multiplicity of the signals; s = singlet, d = doublet, m = multiplet, br m = broad multiplet. Matrix-assisted laser desorption/ionization time-of-flight mass spectrometry (MALDI-TOF-MS) was conducted using a Shimadzu Biotech AXIMA Confidence MALDI mass spectrometer in linear (positive) mode, referencing against poly(propylene glycol), M_n = 4.0 kg mol^{-1}. 50 µL of polymer solution (1 mg mL^{-1} in THF) was mixed with 50 µL of a 10 mg mL^{-1} solution of the matrix (dithranol) in THF. A drop of this solution was spotted onto a MALDI plate and the solvent allowed to evaporate at room temperature. Elemental compositions of carbon, hydrogen and sulfur atoms were measured using a Flash 2000 Organic Elemental Analyser (Thermo Scientific). Elemental composition of bromine was determined by titration against silver nitrate using an 888 Titrando (Metrohm). Nominal and high resolution electrospray mass spectrometry were carried out using SQD2 and QTOF Spectrometers (Waters). Infrared spectroscopy was conducted using a Nicolet iS5 (Thermo Scientific) with iD5 ATR accessory. Gel permeation chromatography (GPC) was conducted in THF using a Viscotek GPCmax VE2001 solvent/sample module with 2 × PL gel 10 µm MIXED-B + 1 × PL gel 500A columns, a Viscotek VE3580 RI detector and a VE 3240 UV-Vis multichannel detector. The flow rate was 1 mL min^{-1} and the system was calibrated with narrow PDI polystyrene standards in the range of 0.2 – 1,800 kg mol^{-1} from Polymer Laboratories. The analysed samples contained *n*-dodecane as a flow marker. UV-Vis absorption spectra and optical densities were recorded on a Varian Cary 5000 UV-Vis-NIR spectrophotometer and photoluminescence spectra were recorded on Cary Eclipse Fluorescence Spectrophotometer. Slow additions were performed using a 205S Watsons-Marlow peristaltic pump.

THF was distilled over sodium/benzophenone and all other anhydrous solvents were purchased from Sigma-Aldrich or Alfa Aesar and used as received. All other reagents were purchased from Sigma-Aldrich, Fisher Scientific, Alfa Aesar or Acros and used has received. Column chromatography was performed using silica gel (60 Å, 230−400 mesh). Petroleum ether refers to the fraction obtained at 40-60 ˚C. All reactions were carried out using standard Schlenk techniques under argon, unless stated otherwise. Degassed solvents were prepared either by purging with argon/nitrogen or by freeze-pump-thaw (three times) for reactions involving organometallic reagents.

General procedure for *in-situ* NMR REMP experiment of monomer (M1 and M2) with catalyst 1.

M (30 mg, 10 *eq*.) and **G** (1 *eq*.) were added into separate vials and transferred into an argon filled glovebox. **G** was dissolved in benzene-d_6 ([**M**] = 100 mM) and the solution was transferred to the vial containing **M** and mixed until homogenous. The solution was transferred into a Young's NMR tube, sealed, removed from the glovebox and kept in an ice bath. The first ^{1}H NMR spectrum for t=0 was recorded at 25 °C. Then the spectrometer probe was set at 60 °C and the NMR spectra were recorded at every 5 min intervals throughout the REMP (At the end of the reaction mixture was quenched with ethyl vinyl ether followed by purification *via* precipitation into methanol/Celite plug followed by extraction of the polymer with chloroform. These trace linear polymer contaminants were successfully removed by silica column chromatography by eluting with toluene and hexane (20/80) solvent mixture.

General procedure for the ROMP of M1 with catalyst G3:[1] In an argon filled glovebox cyclophanediene **M1** and **G3** were added to a vial with a stirrer bar, followed by deoxygenated, anhydrous THF ([**M1**]$_I$ = [0.1]$_I$). The vial was sealed, removed from the glovebox, wrapped in foil and mixed at room temperature for 10 minutes. The reaction was placed in a preheated oil bath at 40 °C and stirred until complete monomer conversion. The reaction was cooled to room temperature and deoxygenated ethyl vinyl ether was added and stirred at room temperature for 2 hours. The reaction was precipitated into a short methanol/Celite column, washed with methanol and the polymer extracted with chloroform. After evaporation of the solvent linear poly(*p*-phenylenevinylene) polymers were isolated as green films. Linear PPV$_{10}$ shown below has been prepared following this general procedure; M_n^a =4.4 kD, M_n^b = 5.5 kD, *Đ*=1.28.

(^{a}Calculated from the [M1]/[G3] ratio, including expected end groups, ^{b}determined by GPC with RI detection (calibrated against narrow Đ*m* polystyrene standards).

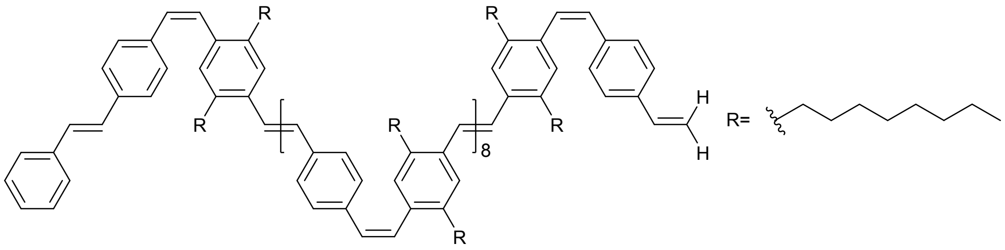

*l*PPV$_{10}$

REMP of alkyl monomer M1 with catalyst 1:

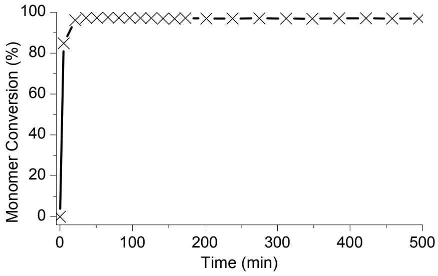

Figure S-1: REMP of monomer **M1** with catalyst **1** – monomer conversion

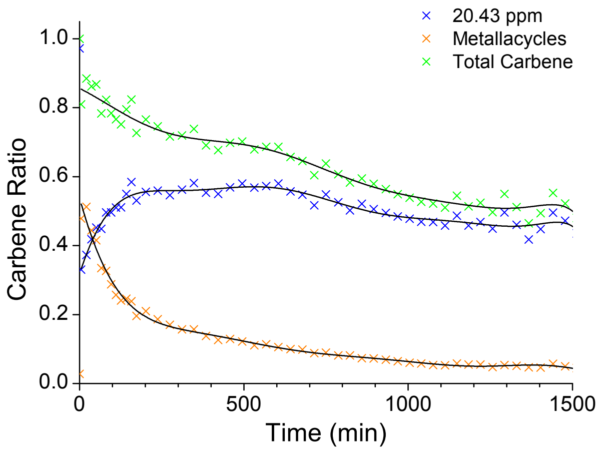

Figure S-2: REMP of monomer **M1** with catalyst **1** -change in carbene integration with time

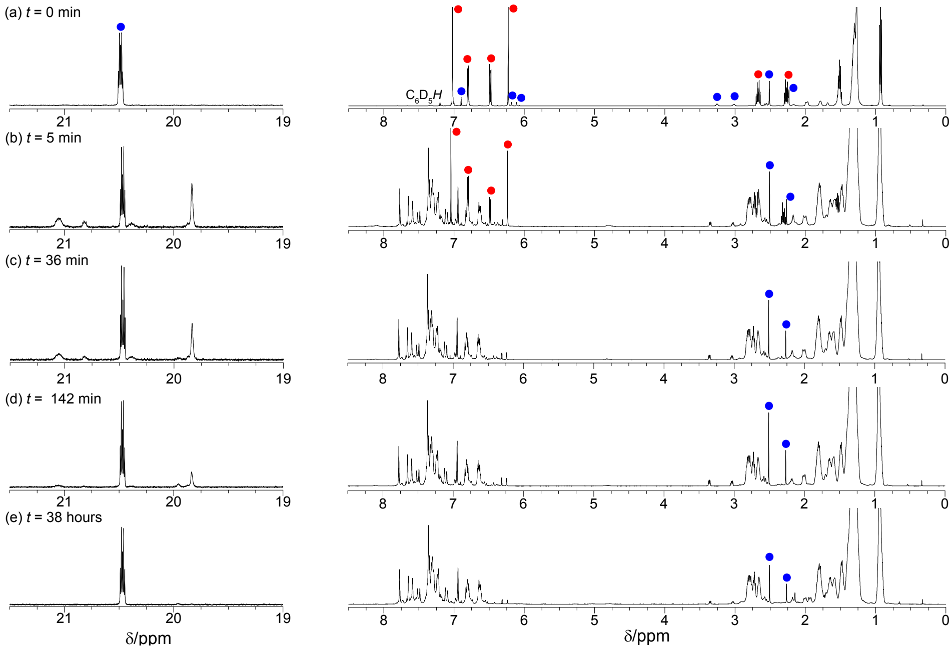

Figure S-3: Time dependent stack *in-situ* NMR of REMP plot for dialkyl paracyclophanediene monomer **M1** using catalyst **1**.

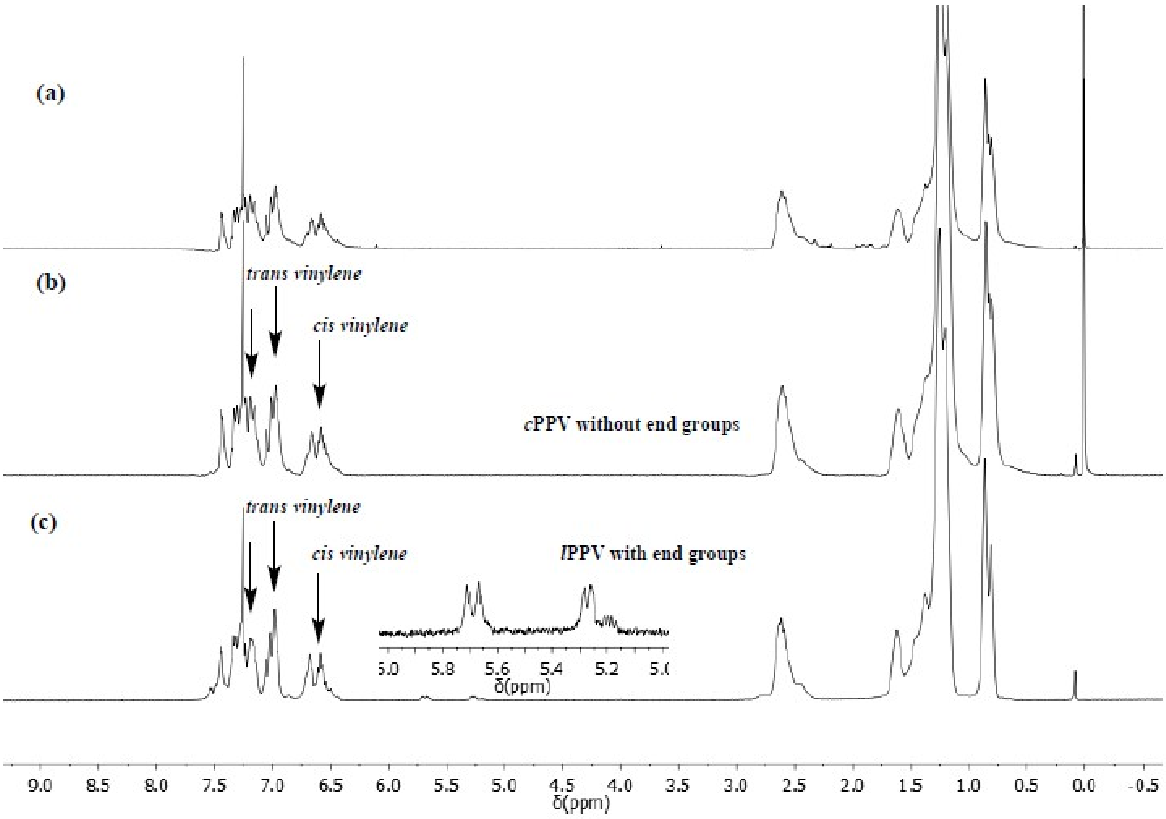

Figure S-4. ^{1}H NMR spectra of dialkyl *c*PPVs; (a) crude polymer sample, (b) isolated polymer after precipitation into methanol, (c) ^{1}H NMR spectrum of dialkyl *l*PPV_{10} in $CHCl_3$

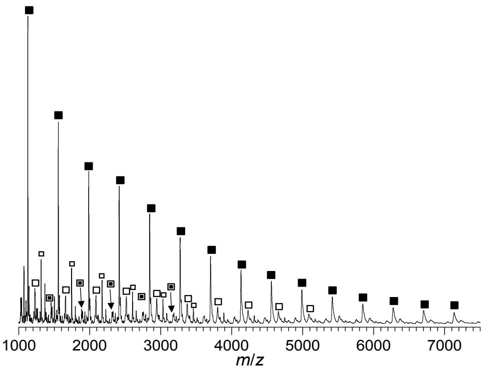

Figure S-5. MALDI-TOF-mass spectrum of polymer from the REMP of **M1** obtained after precipitation into methanol, mass region 1000-7500Da. **○, ◉ and ⅄** correspond to linear polymers and the presence of the linear polymer ◉ and ⅄ shows that secondary metathesis is occurring in the reaction; □ (not identified).

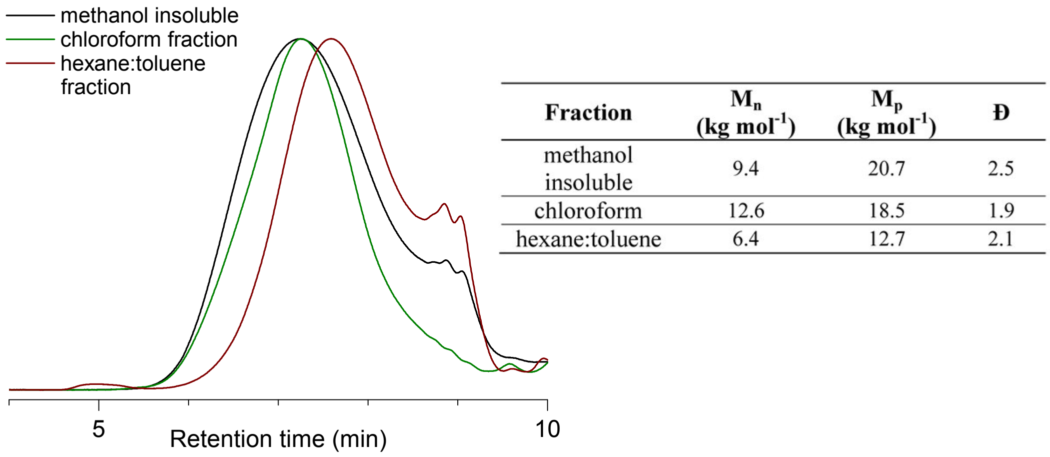

Fraction	M_n (kg mol^{-1})	M_p (kg mol^{-1})	Đ
methanol insoluble	9.4	20.7	2.5
chloroform	12.6	18.5	1.9
hexane:toluene	6.4	12.7	2.1

Figure S-6. GPC chromatograms of polymer from the REMP of monomer **M1** and summarised molecular weight data for each fraction (calibrated against narrow Đ PS standards).

Table S1: Optimsation of REMP of dialkyl cyclophanediene monomer **M1** with catalyst **1**

Sl.no	[M]/[C]	Solvent	°C	[M]	Time (min)	M_n (kgmol^{-1})[cal.]	M_n (kg mol^{-1})[obs.]	M_p (kg mol^{-1})[obs.]	*Đ*m[obs.]
1	10	benzene	60	0.1	90	4.3	9.4	20.7	2.5
2	10	benzene	60	0.1	90	4.3	7.8	18.5	2.9
3	10	benzene	60	0.05	90	4.3	14.7	24.0	1.8
4	10	benzene	60	0.025	90	4.3	8.2	16.3	2.5
5	20	benzene	60	0.1	90	8.6	15.0	33.0	2.4
6	20	benzene	60	0.05	90	8.6	13.0	26.2	2.3
7	20	benzene	60	0.025	90	8.6	15.0	33.3	3.4
8	10	THF	50	0.25	180	4.3	13.4	20.9	1.8
9	10	THF	50	0.25	180	4.3	8.8	14.6	1.9

Obs. Determined by GPC with RI detector (calibrated against narrow $Đ_m$, PS standards).

Table S2: Calculated and observed masses of cyclic species in the MALDI-TOF-mass spectrum of polymer from REMP of monomer **M1** with catalyst **1**

		X_n				
**Species**	**Mass (Da)**	**5**	**6**	**7**	**8**	**9**
**cPPV1**	Calc. (*m/z*)[a]	2143.5	2572.1	3000.8	3429.5	3858.2
	Obs. (*m/z*)	2143.3	2572.0	3000.7	3429.5	3858.2
	Δ	- 0.2	- 0.1	- 0.1	+ 0.0	+ 0.0
**cPPV2**	Calc. (*m/z*) [a]	2245.6	2674.3	3103.0	3531.7	3960.4
	Obs. (*m/z*)	2245.4	2673.8	3103.2	3531.6	3960.0
	Δ	- 0.2	+ 0.5	+ 0.2	-0.1	-0.4
**cPPV3**	Calc. (*m/z*) [a]	2470.0	2898.7	3327.4	3756.1	4184.8
	Obs. (*m/z*)	2469.4	2898.2	3327.4	3755.7	4185.2
	Δ	-0.6	-0.5	0.0	-0.4	+0.4

[a]Calculated from the average isotopic mass of cyclophanediene monomer **M1** ($C_{32}H_{44}$) to be 428.69.

REMP of monomer M2 with catalyst 1:

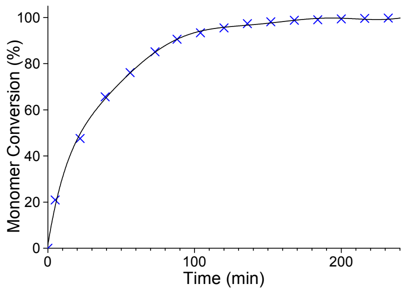

Figure S-7: REMP of monomer **M2** with catalyst **1** – monomer conversion

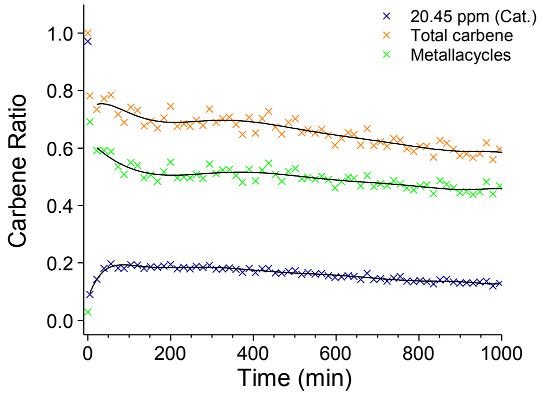

Figure S-8: REMP of monomer **M2** with catalyst **1** - Change in carbene integration with time

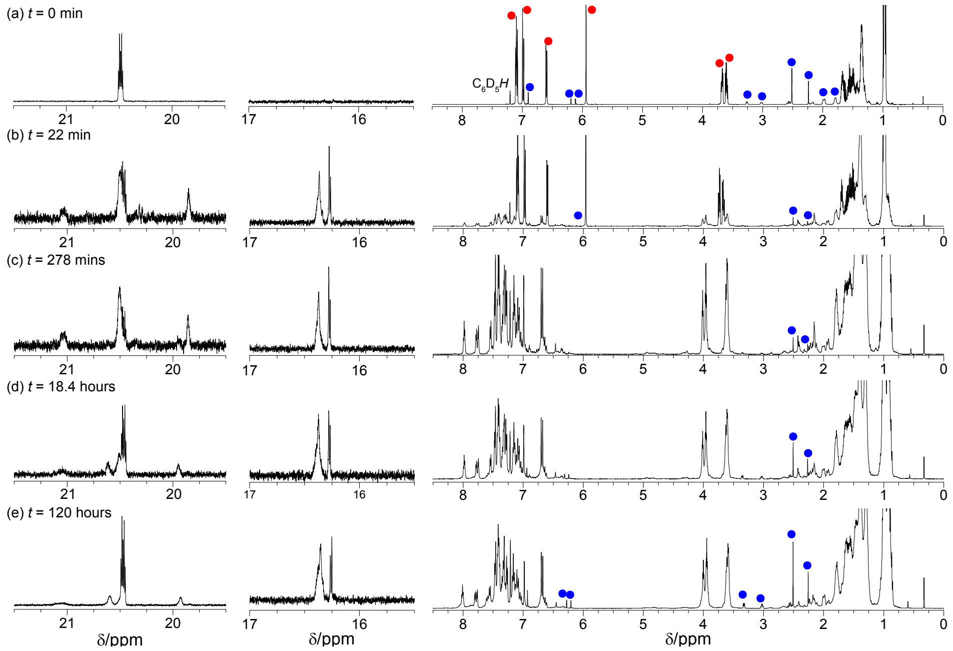

Figure S-9: Time dependent stack of *in-situ* NMR of REMP plot for dialkoxy paracyclophanediene **M2** using catalyst **1**.

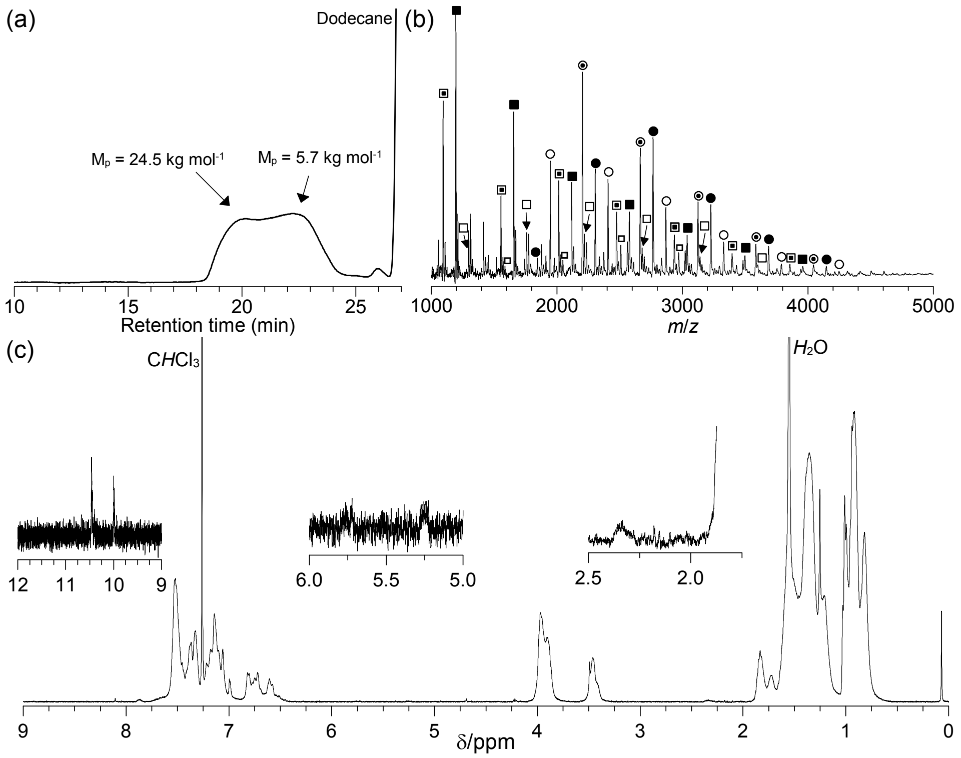

Figure S-10. Characterisation of polymer dialkoxy *c/l* PPVs, obtained after extraction with hexane; (a) GPC chromatogram, (b) MALDI-TOF-mass spectrum shows three major linear polymer series; O, O and ⅄ and three major cyclic series; ℘, ⅋ and ·, mass region 1000-5000 Da and (c) ^{1}H NMR spectrum.

Sample preparation and experimental setup for photophysical and single-molecule characterisation

Sample preparation

For single-molecule experiments the *l***PPV** and *c***PPV** were dispersed in 100 nm thick spin-coated films of poly(methyl methacrylate) (PMMA) at the concentration of 10^{-9}M. For bulk spectroscopic characterisation the PPVs were dispersed in a free-standing poly(ethyl methacrylate) (PEMA) film at an ensemble concentration. The free-standing film is prepared by drop-casting the PPV doped polymer solution on a substrate, drying at ambient conditions and peeling off the film.

Experimental setup

Single-molecule experiments were performed using an inverted fluorescence microscope (Olympus IX71, UPlanFLN100xO2 oil-immersion lens) with a 375 nm diode laser (PicoQuant) excitation and an electron multiplication (EM) CCD camera detection (Andor iXon). Fluorescence spectra of single molecules were measured using an imaging spectrograph (Bunkou Keiki CLP-50) inserted between the microscope and the EM-CCD camera. For the absorption (excitation) anisotropy measurements the linear polarization of the excitation laser was periodically switched between two orthogonal polarization directions with an opto-electrical modulator (Conoptics) and fluorescence images were recorded for each polarization direction. For the fluorescence anisotropy experiments, the excitation laser was circularly polarized and the single-molecule fluorescence image was split using a Wollaston prism into *s*- and *p*-polarized images. Samples were purged with N_2 gas during the experiments to suppress photobleaching. Fluorescence spectra and spectrally resolved fluorescence lifetime in the stretched bulk films were recorded using a fluorescence spectrometer (Jasco, FP-6200) and a compact fluorescence lifetime spectrometer (Hamamatsu, Quantaurus-Tau), respectively. Time-dependent density functional theory (TD-DFT) calculations were performed using the Gaussian 09 program package [Frisch, M. J. *et.al*., Gaussian 09, Revision D.01, Gaussian, Inc., Wallingford, CT, **2009**] with the B3LYP functional using 6-31G(d,p) basis sets.

Single-molecule absorption polarisation anisotropy

The absorption anisotropy for each single molecule was evaluated by analyzing the emission intensities I_s and I_p corresponding to two orthogonal linear polarizations s and p of the excitation laser and calculating the degree of the anisotropy as $a_A = (I_s - I_p)/(I_s + I_p)$. The distributions of a_A for both *l***PPV** and *c***PPV** obtained for a statistical ensemble of 276 (*l***PPV**) and 136 (*c***PPV**) single molecules (Figure 4a,b) are centered at 0 values and most of the data points are located between -0.5

and 0.5. The distribution of the *c***PPV** is slightly sharper. The measured absorption anisotropy distributions are results of average conformations of the *l***PPV** and *c***PPV** chains. To reconstruct the conformations from the experimental data we performed numerical simulations. Assuming in analogy with other phenylene vinylene polymers that the conjugation length is approximately 6 monomer units the chain is tentatively modelled using 6 conjugated segments, each represented by a transition dipole moment. We have verified by the TD-DFT calculations that the transition dipole moments in the regularly alternating cis/trans chain are oriented approximately along the main axis of the polymer chain, as shown in **Figure S-11**. In solution, both species can undergo *cis/trans* photoisomerization and possibly thermally induced cis/trans isomerization. The resulting trans-segments have transition dipoles again oriented along the polymer chain. In any case, the chains can be represented by 6 head-to-tail aligned transition dipole moments (rods). Orientation of the absorption transition dipole moment with respect to the direction of the two orthogonal polarizations corresponds to the spatial orientation of the 6-monomer conjugated segment. In the model for the *l***PPV**, the first segment lies in the sample plane (*s*-*p* plane) at a random angle from the *s* polarization direction. Projection of the angle between the second and the first segment onto the sample plane is denoted ϕ, the angle of the second segment from the plane is θ. The angle ϕ is allowed to vary randomly within the range $\Delta\phi$. For all following segments, the ϕ is projected on a plane defined by the preceding two segments and the angle θ is defined from this plane. For a given conformation of a chain, the components of the absorption along the s and p directions A_{si} and A_{pi} are calculated for each segment i, and the anisotropy of the chain is calculated as $a_A = \left(\sum_i A_{si} - \sum_i A_{pi}\right) \Big/ \left(\sum_i A_{si} + \sum_i A_{pi}\right)$. In the simulations, we first set $\theta = 0$ and chose a set of the values of ϕ, $\Delta\phi$. The values of the angle of the first segment from the s polarization direction were generated randomly, the corresponding a_A were calculated for these randomly generated angles and the values a_A were plotted in a histogram. The sets of values ϕ, $\Delta\phi$ were then changed systematically in defined increments and for each set a histogram of the a_A was constructed, as shown in **Figure S-12**. These simulated histograms of the projection of the chain conformation onto the sample plane were compared with the experimentally measured ones and the most probable projection of the conformation of the *l***PPV** chain was selected, as shown in Figure 4a. The values of the angles for this conformation are $\phi = 30^{o}$, $\Delta\phi = 5^{o}$, and the chain conformation is schematically shown above the Fig. 4a. Further, for this most probable projection of conformation we systematically increased the angle θ in increments of 5° and for each value of θ recalculated the corresponding histogram of the a_A values. The results indicated that the shape of the histogram and its correspondence with the experimental values do not change significantly for of θ between 0 and 20°.

For the cyclic polymer *c***PPV**, the simulation has to account for the fact that the end of the last segment has to coincide with the beginning of the first segment. To satisfy this condition, the model

as described above was used for the first 3 segments, and the remaining 3 segments were constructed as a mirror image of the first three. The simulations, the calculation of the anisotropy a_A and the statistical analysis were done in the same way as for the linear *l***PPV**. The most probable projection of the conformation of the *c***PPV** onto the sample plane has the values of the angles of $\phi = 60^o$, $\Delta\phi = 10^o$ (Figure 4b and above), and the shape of the histogram again does not change significantly for of θ between 0 and 20°.

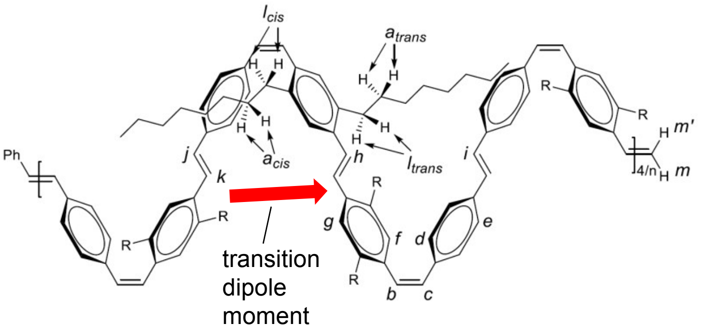

Figure S-11. Orientation of a transition dipole across a *cis*-conformation obtained from DFT calculations

Single-molecule fluorescence polarisation anisotropy

For each single chain the s and p polarized fluorescence intensities I_s and I_p were analyzed and the degree of the anisotropy was calculated as $a_F = (I_s - I_p)/(I_s + I_p)$. The distributions of a_F for both *l***PPV**V and *c***PPV** obtained for a statistical ensemble of 108 (*l***PPV**) and 145 (*c***PPV**) single molecules are shown in Figure 4 c,d. Unlike the absorption anisotropy, the fluorescence anisotropy is markedly different between the cyclic and linear polymers. The a_F distribution for *c***PPV** has a broad peak centered at 0 and spans values from -1 to 1. The distribution for *l***PPV** shows a bi-modal structure with peaks at approximately -0.4 and 0.4, a minimum at 0 and with values spanning the whole range from -1 to 1.

The measured distributions of a_F were reproduced by numerical simulations. The simulations were done in a similar way as for the absorption anisotropy but with the assumption that the emission originates from a limited number of conjugated segments due to efficient energy transfer and exciton localization. The a_F was calculated for the emission from the limited number of segments whose orientations were determined from the simulations. For the whole set of all conformation projections (ϕ, $\Delta\phi$ values) the number of emitting segments were systematically decreased to 5, 4, 3 and 2. An example of the simulated histograms for 3 emitting conjugated segments is shown in **Figure S-13**. In each chain the actual emitting segments were chosen randomly. Statistical comparison of the simulated and measured distributions showed that the experiments are best reproduced with the emission from 3 conjugated segments for both *l***PPV** and *c***PPV** (Figure 4c,d). The corresponding most probable projection of the conformation of the *l***PPV** (Figure 4c, above) is given by the angles

ϕ = 20°, $\Delta\phi$ = 5°, the projection of the conformation of the *c***PPV** is characterised by the angles ϕ = 70°, $\Delta\phi$ = 15° (Figure 4d, above). These values are very close to those obtained from the absorption anisotropy measurements and confirm the validity of this approach and reliability of the reconstructed conformations. As in the case of absorption anisotropy, the shapes of the simulated histograms and their correspondence with the experimental distributions do not change significantly for the values of θ between 0 and 20°.

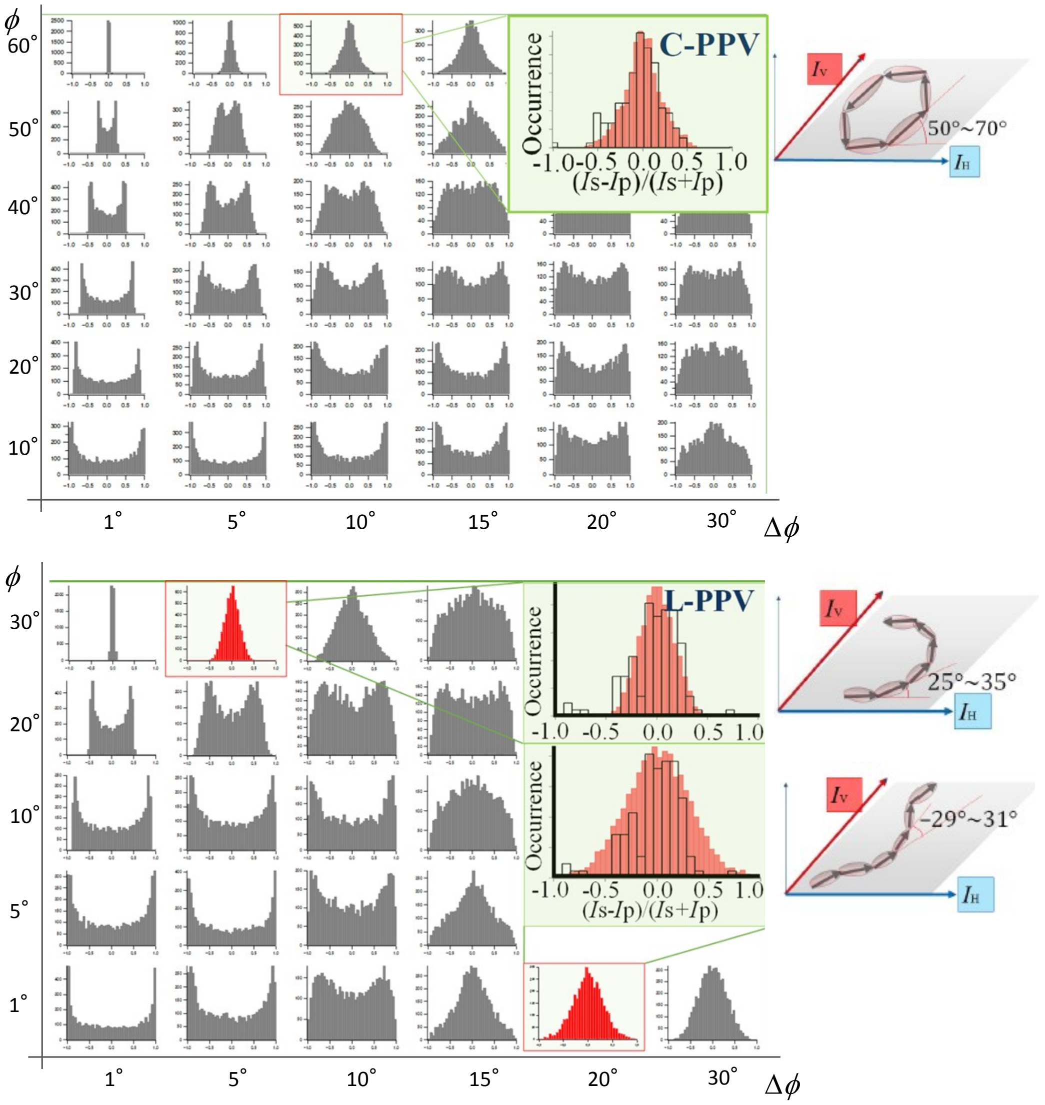

Figure S-12. Simulated histograms of absorption anisotropy for single chains of *c***PPV** (top) and *l***PPV** (bottom). The insets show comparison with experimentally obtained histograms. The illustrations on the right show schematically the corresponding conformations.

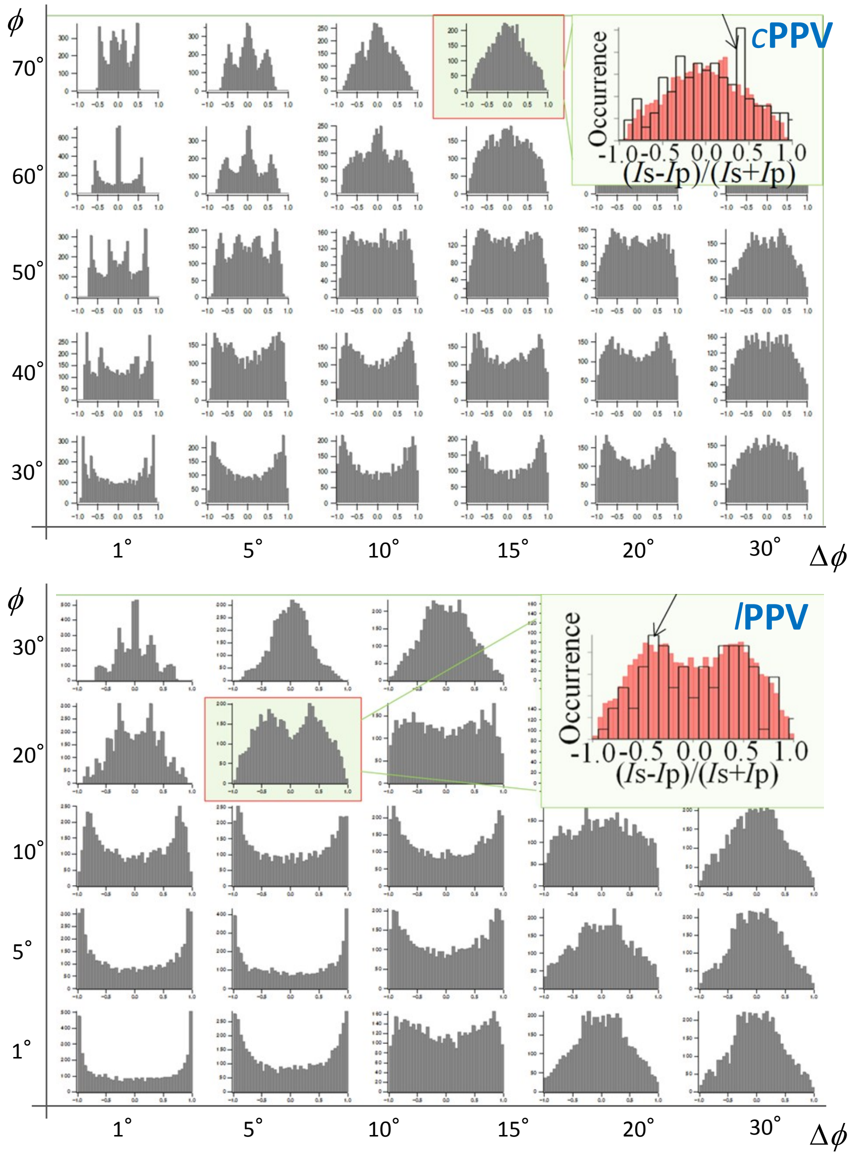

Figure S-13. Simulated histograms of fluorescence anisotropy for single chains of *c***PPV** (top) and *l***PPV** (bottom), considering 3 randomly-distributed emitting segments. The insets show comparison with experimentally obtained histograms.

Bulk spectroscopic characterisation

To get insight into the nature of the observed spectral features and the conformational differences between the two compounds, the *l***PPV** and *c***PPV** were doped into free-standing PEMA films prepared by drop casting. To induce conformational changes in the PPV and introduce structural defects, the films were heated above glass transition temperature and physically stretched in one direction to about twice of the original length. After cooling back to room temperature bulk fluorescence spectra of the stretched films were measured. The results are shown in **Figure S-14**. Stretching of the films causes blue shift of the main band and an appearance of a short-wavelength shoulder. Both these effects are more prominent for the *c***PPV** compound. The short-wavelength shoulder coincides in wavelength with the short-wavelength shoulder observed on the single-molecule level. Further, we measured spectrally resolved fluorescence lifetimes in the stretched films. In both *l***PPV** and *c***PPV**, the lifetime shows two components, a short one of 0.9 ns (in both compounds) and a long one of 6.8 ns (in *l***PPV**) and of 6.9 ns (in *c***PPV**). Interestingly, both components show different wavelength dependence. As seen in **Figure S-14**, the short component is located primarily in the region of the main fluorescence band whereas the long component is blue-shifted and overlaps more with the short-wavelength shoulder. This large difference in lifetimes between the main band and the shoulder indicates that the origin of the two spectral features is more complex than a simple difference in conjugation length, as assumed from the single-molecule spectral characterization.

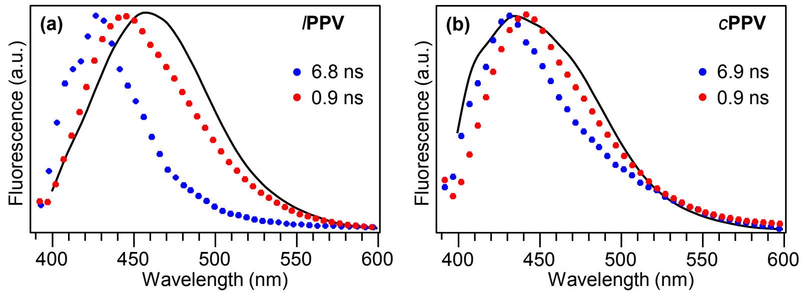

Figure S-14. Ensemble fluorescence spectra of *l***PPV** (a) and *c***PPV** (b) doped into stretched bulk PEMA films (black solid lines). The symbols indicate spectrally resolved components of fluorescence lifetime.

TD-DFT calculations

We attempted to reproduce and interpret the single-molecule and bulk spectroscopic features by TD-DFT calculations. In particular, we tried to reproduce the large distribution of the single-molecule spectral peaks within the main band, and the appearance of the blue shifted shoulder (both on single-molecule and bulk levels) and its increased fluorescence lifetime (on the bulk level). The calculations are based on the assumption that in solution at least part of the cis conformations in the alternating

cis/trans chain undergo *cis/trans* photoisomerisation and possibly thermally induced cis/trans isomerization, resulting in the presence of all-trans segments of different lengths. The calculations were done on linear units containing 8 phenylene vinylene (PV) monomers. A few examples of the results are shown in Figure **S-15**. To simulate part of the polymer chain on which two neighboring cis conformations underwent the isomerization, 8 PV monomers were prepared in all-trans conformation and the conjugation was restricted by rotating and fixing the end phenylene units by 90°. The structure was optimized and energy gap and oscillator strength calculated in the first excited state S_1, Figure **S-15a**. The result was a strongly allowed transition with the oscillator strength of 5.36. To simulate conformational distortions, part of the chain containing 3 PV units was rotated in increments of 10°, as indicated in Figure **S-15b**. An increase of the torsional rotational angle causes an increasing shift of the energy gap to higher energies (blue shift of the spectrum) but does not much effect the oscillator strength which stays on the order of 5. An example of such distorted structure is shown on Figure **S-15b** where rotation by 40° caused a blue shift by 30 nm. Physically, these torsional distortions could be caused by entanglement of the side groups with the chains of the matrix polymer, and this effect could contribute to the large distribution of the single-molecule main band spectral peaks as well as the blue shift upon stretching of the bulk film. Based on these calculation results, we may assign the main fluorescence band in both *l***PPV** and *c***PPV** to longer all-trans segments with different amount of torsional defects. Simulation results of a part of the chain on which only one cis conformation underwent the cis/trans isomerization are shown in Figure **S-15c**. The energy gap of this shorter trans-segment was shifted to the blue by 32 nm and the oscillator strength dropped to 3.75. These values show that such shorter segments could also contribute to the main fluorescence band in both species.

Calculation of the alternating cis/trans 8 PV unit (not shown) results in very small oscillator strength (0.015) and an energy gap shifted to the red by more than 300 nm. It is unlikely that these conformations even if they were present would contribute to the measured fluorescence spectra. On the other hand, rotation of half of the 8 PV unit along a single bond in the central cis state (as indicated in Figure **S-15d**) leads to a blue shift with respect to the undistorted all-trans unit and to an oscillator strength on the order of 1.4 – 2.0. As an example, the rotational angle of 120° (Figure **S-15d**) causes a blue shift of 77 nm and an oscillator strength of 1.62. This amount of blue shift would correspond to the short-wavelength shoulder observed both on single-molecule and bulk levels, and it is plausible that torsionally distorted cis conformational states could contribute to the emission in this spectral range. In addition, the considerably smaller oscillator strength of these states would result in longer radiative lifetime and explain the longer fluorescence lifetime observed in the short-wavelength shoulder for both *l***PPV** and *c***PPV** on the bulk level.

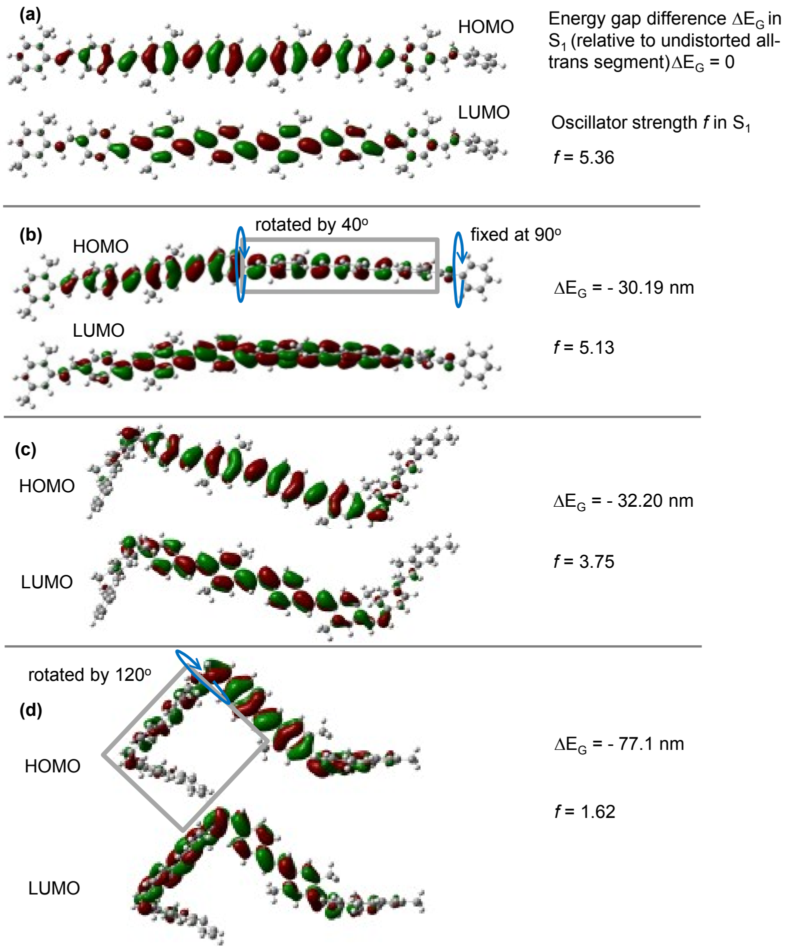

Figure S-15. Results of TD-DFT calculations of an optimized all-trans 8 PV unit (a), of an all-trans unit with torsional distortion (b), of a unit with the central cis state isomerized (c), and of an alternating *cis/trans* unit with torsional distortion (d).

References

1. B. J. Lidster, D. R. Kumar, A. M. Spring, C.-Y. Yu and M. L. Turner, *Polym. Chem.*, 2016, **7**, 5544-5551.
